# High-Q Fabry–Pérot Micro-Cavities for High-Sensitivity Volume Refractometry

**DOI:** 10.3390/mi9020054

**Published:** 2018-01-31

**Authors:** Noha Gaber, Yasser M. Sabry, Mazen Erfan, Frédéric Marty, Tarik Bourouina

**Affiliations:** 1 Université Paris-Est, ESIEE Paris, ESYCOM EA 2552, 93162 Noisy-le-Grand, France; mazen.erfan@esiee.fr (M.E.); frederic.marty@esiee.fr (F.M.); tarik.bourouina@esiee.fr (T.B.); 2 Center for nanotechnology, Zewail City of Science and Technology, Sheikh Zayed District, 6th of October City 12588, Giza, Egypt; ngaber@zewailcity.edu.eg; 3 Faculty of Engineering, Ain-Shams University, 1 Elsarayat St., Abbassia, Cairo 11517, Egypt; yasser.sabry@eng.asu.edu.eg

**Keywords:** Fabry–Pérot cavity, optical micro cavity, on-chip refractometer, refractive index measurement, stable optical resonator, optofluidic sensor, lab-on-a-chip

## Abstract

This work reports a novel structure for a Fabry–Pérot micro cavity that combines the highest reported quality factor for an on-chip Fabry–Pérot resonator that exceeds 9800, and a very high sensitivity for an on-chip volume refractometer based on a Fabry–Pérot cavity that is about 1000 nm/refractive index unit (RIU). The structure consists of two cylindrical Bragg micromirrors that achieve confinement of the Gaussian beam in the plan parallel to the chip substrate, while for the perpendicular plan, external fiber rod lenses (FRLs) are placed in the optical path of the input and the output of the cavity. This novel structure overcomes number of the drawbacks presented in previous designs. The analyte is passed between the mirrors, enabling its detection from the resonance peak wavelengths of the transmission spectra. Mixtures of ethanol and deionized (DI)-water with different ratios are used as analytes with different refractive indices to exploit the device as a micro-opto-fluidic refractometer. The design criteria are detailed and the modeling is based on Gaussian-optics equations, which depicts a scenario closer to reality than the usually used ray-optics modeling.

## 1. Introduction

On-chip sensing devices are receiving increasing attention due to their appealing features of compact size, rapidity, small volume required of the samples under test, and their ability to be mass-produced, which greatly reduces the cost. They take advantage of the fast-growing techniques for micro and nano fabrication, which enables scaled-down benchtop instruments and the implementation of the same functional devices on-chip. Refractometers are popular instruments found in many labs, used, for instance, in measuring the refractive index (RI) of dielectric materials, or determining the concentration of some solutions. There have been several attempts to implement various techniques of refractometry on-chip [1,2,3]. This includes different type of resonators, such as micro-ring, micro-toroids and micro-disc, micro-sphere, and photonic crystal [4,5,6,7,8,9]. A novel three rolled-up microtubes structure has also been reported [10]. These resonator types exhibit high-quality factor resonance, but they can enable only surface refractometry, not volume refractometry. Surface refractometry is preferred when high sensitivity is the maindemand for the refractometer; while volume refractometers are preferred in applications requiring a high depth of interaction between the light and the sample. For instance, for penetration through large biological cells that are too large for the small evanescent tail of surface waves to penetrate, a volume refractometer must be used. Additionally, in applications where surface contamination imposes some risk to accurate detection, avoiding surface refractometry is preferred.

One attractive volume refractometry technique is the Fabry–Pérot (FP) optical cavity. It can be easily implemented on-chip, as it simply consists of two optical mirrors separated by the designed cavity length (*d*). When the liquid under test of refractive index (*n*) passes inside the cavity, its resonance peaks shift in wavelength (*λ*) depending on that value. It is convenient to have sharp and highly selective resonance peaks for accurate measurements; therefore, it is preferable to have a resonator with high quality factor (*Q*). Also the sensitivity and range should be high, which are the most attractive features of a refractometer. Several on-chip FP cavities with straight mirrors have been reported [11,12,13]. However, the highest reported *Q* factor is only 400 in such cases [11], as the straight mirror shape is mismatched with the wave front of the Gaussian light beam. When using curved mirrors and a micro tube holding the analyte, the Gaussian beam (GB) is better confined and higher *Q* factors up to 2800 can be achieved in the case of non-absorbing liquids [14]. On the other hand, sensitivity is not very high. The reason behind that has been proven to be the low filling factor of the analyte, as it does not fill the entire cavity [15]. In previous studies, the highest reported sensitivity was 1392 in the case of the sample occupying the whole cavity [16]; however, this comes at the expense of the quality factor, and hence the peaks are not very sharp. It should be kept in mind that the stated values do not provide an accurate quantitative comparison for the refractometer performance, as the sensitivity value depends on the refractive index and absorption of the analyte used, which is different in each reported work. Still, it can provide an approximate evaluation.

**Figure 1:**
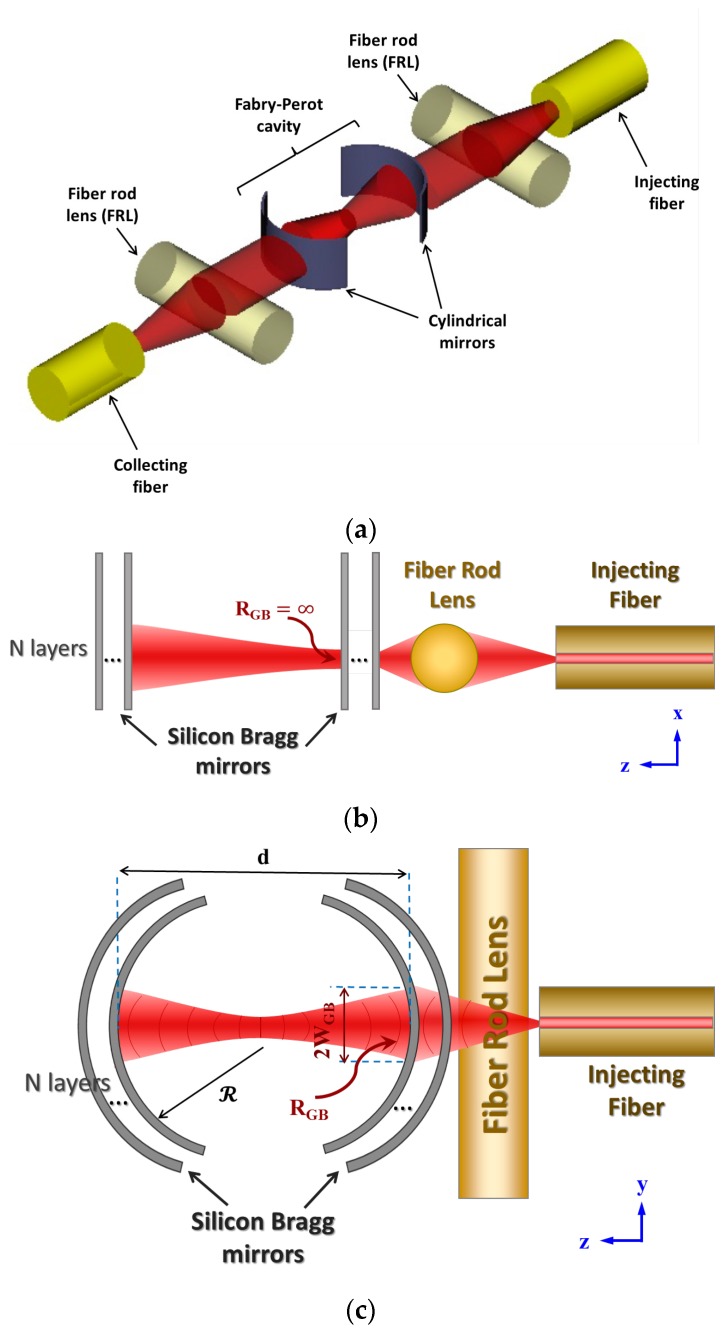
(**a**) 3D schematic of the proposed Fabry–Pérot (FP) cavity. Two-dimensional schematic of the proposed FP cavity with the design geometries for: (**b**) Side view (x-z plan); (**c**) Top view (y-z plan).

In this article, a novel FP microcavity structure is proposed, achieving both a high *Q* factor resonator and a high-sensitivity refractometer. The proposed optical structure is schematically depicted in Figure 1a. It employs cylindrical Bragg mirrors forming the FP cavity to confine light in the lateral plane, while external cylindrical lenses—implemented by a fiber rod lens (FRL)—are used to confine light in the vertical plane before it enters the FP cavity. By having the FRL outside the cavity, the loss due to light reflection from its surface does not deteriorate the quality factor of the resonator.

Another advantage of the proposed structure is that the space between the mirrors is free, allowing the liquid to fill the whole cavity, which enables the light bouncing inside the cavity to pass through the analyte along its entire trip. This can be understood from the formula describing the sensitivity *δλ*/*δn*:

(1) $$\frac{\delta \lambda }{\delta n}=\lambda \frac{d_{\mathrm{liquid}}}{L_{\mathrm{eff}}}$$

Where *d*_liquid_ is the path length inside the liquid, and *L*_eff_ is the effective total path length of the light. From Equation (1), it can be deduced that the highest sensitivity is attained by having the maximum ratio (*d*_liquid_/*L*_eff_). The *L*_eff_ of the cavity is expected to be a bit larger than (*d* × *n*), as the employed mirrors are distributed Bragg mirrors; hence, the light reflection does not actually happen at the interior surface, but rather somewhere inside the mirror’s bilayers.

## 2. Design

As indicated above, good confinement of the light beam inside the cavity requires matching between the wave front of the light beam (Gaussian in this case) and the mirror shape. Otherwise, the beam suffers from expansion after few round trips inside the cavity and escapes from the micro-mirrors’ borders, which is called “diffraction loss”. This loss reduces the quality factor of the resonator, as happens in the widely employed straight mirrors. For ideal matching, these mirrors should have a paraboloid curvature in 3D that matches the GB wave form; hence, the GB will reflect on itself, producing almost a self-sustained mode inside the cavity. However, such curvature in 3D is very challenging to achieve by standard microfabrication techniques [17,18]. To overcome this challenge, the curvature is decoupled in the horizontal and vertical plans; and partial matching with the wave phase front is employed in each plan, as detailed below.

The GB optics models are adopted in the design for better accuracy, instead of the simpler ray model used previously in modeling closely related structures [14,19]. The basic equations describing the intensity of the GB are stated in Equations (2)–(5) in the case of a homogeneous medium of refractive index *n* [20]:

(2) $$I\left(x,y,z\right)=I_{0}{\left[\frac{W_{0}}{W_{\mathrm{GB}}\left(z\right)}\right]}^{2}\exp\left[-2\left(\frac{x^{2}+y^{2}}{{W_{\mathrm{GB}}}^{2}\left(z\right)}\right)\right]$$

(3) $$W_{\mathrm{GB}}\left(z\right)=W_{0}\sqrt{1+{\left(\frac{z}{z_{0}}\right)}^{2}}$$

(4) $$R_{\mathrm{GB}}\left(z\right)=z\left[1+{\left(\frac{z_{0}}{z}\right)}^{2}\right]$$

(5) $$z_{0}=\frac{\pi {W_{0}}^{2}n}{\lambda }$$

where *W*_GB_(*z*) and *R*_GB_(*z*) are the beam width and wave front radius of curvature, respectively. *z*_0_ is the Rayleigh range, *W*_0_ is the beam waist radius, and the beam waist diameter 2*W*_0_ is also called the spot size.

As described above, the structure consists of an FP resonator with curved Bragg mirrors of multi-bilayers of silicon/air and external FRLs. Different numbers of layers (N) are used—from two to five bilayers. The reflectivity of the mirror increases with increasing the number of bilayers, but also the insertion loss increases. The FRL is simply a piece of striped fiber which has a diameter of 125 µm and refractive index of 1.467. Figure 1b,c show schematics of the side and top views for the proposed novel structure. The vertical plan and horizontal plan are decoupled in the design for easier analysis. A cleaved fiber with mode field diameter (MFD) of 10.4 ± 0.8 µm at 1550 nm is used for injecting light. The injected light beam can then be modeled as a GB with beam waist radius *W*_0_ = 5.2 µm. For the x-z plan (whose schematic is presented in Figure 1b), the FRL transforms this GB into another beam with *W*_0_ = 4.3 µm, whose beam waist is located at a distance of about 73 µm from the FRL. The beam transformation is calculated using the ABCD law [21]. As there is a spacing between the FRL and the Bragg mirror of about 30–55 µm (due to the margin of the FRL groove), the beam waist is located somewhere inside the entrance mirror. Recalling that the wave front radius of curvature of the Gaussian beam (*R*_GB_) at the beam waist is ∞, that achieves good matching between the strait mirror shape and the light beam shape, enhancing the light coupling into the cavity.

For the y-z plan (Figure 1c), the FRL in this plan acts as a simple sheet of glass, but the beam propagates through it, changing its parameters and experiencing a partial reflection at the interfaces. These effects are calculated through the ABCD law, and the beam radius at the cavity entrance *W*_GB_ is determined to be about 12.53 µm. To acquire good coupling of light into the cavity the fundamental resonant mode inside the cavity is better to have the same beam width at the entrance mirror. This fundamental resonance mode is a GB whose wave front at the mirrors matches the mirror curvature (i.e., *R*_GB_ = ℛ), and its beam waist is located at the middle of the cavity. The cavity length (*d*) is also designed to have the same curvature value (i.e., *d* = ℛ, for the best confinement of light inside the cavity. In this case, the beam radius at the mirrors has its minimum value, and the resonator is symmetrically confocal [21]. This implies that the mirrors are at distance *z*_0_ from the cavity center that hosts the beam waist, and the beam radius at the mirror positions *W*_GB_ = $\sqrt{2}$*W*_0_. As stated above, *W*_GB_ = 12.53 µm; then *W*_0_ = 8.86 µm. By substituting in Equation (5), *z*_0_ = 159 µm, and then ℛ = *d =* 318 µm. Noting that the optical length of the cavity also depends on the analyte refractive index *n*, several cavities with different physical lengths *d =* 318 µm/*n* are implemented on the same chip to account for that effect. Different values for *n*, corresponding to various liquids, are selected.

## 3. Experimental

**Figure 2:**
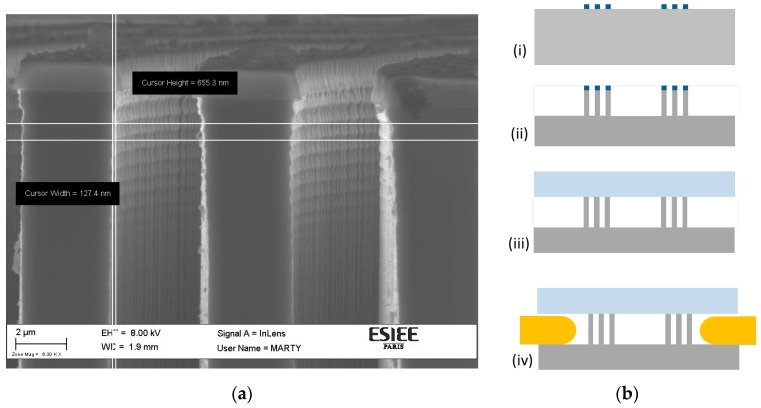
(**a**) A scanning electron microscope (SEM) image of the cross section of the Bragg micro-mirror layers indicating the aspect ratio-dependent scalloping attenuation (ARDSA) effect and the associated reduction of scalloping roughness. (**b**) Simplified fabrication sequence of the volume refractometer: (i) Patterning of single crystal silicon wafer with thermal oxide, (ii) transfer into silicon by deep reactive ion etching (DRIE), (iii) bonding of the glass cap, (iv) inserting the input and output optical fibers from the two sides.

For chip fabrication, single-crystalline silicon with (100)-orientation and a p-type doping level of 10^15^ atoms/cm^3^ was used. A 400 nm-thick thermal oxide was formed and patterned through a lithography step followed by fluorinated plasma etching of the oxide. The oxide patterns were then transferred into the silicon bulk by deep reactive ion etching (DRIE) using a Bosch process involving SF_6_ and C_4_F_8_ for the alternated etching and passivation steps. In order to minimize optical loss and to fulfill the Bragg mirror requirements, the latter etching step is critical. It was optimized with respect to critical dimension loss, the smoothness of the sidewalls, and verticality, with better than 0.1° accuracy. A scanning electron microscope (SEM) image of the cross-section of the Bragg micro-mirror layers is presented in Figure 2a, showing the superior quality of the fabricated mirrors’ walls about 5 µm from the top surface of the silicon substrate. The scalloping effect associated with the DRIE process was noticeable only within the top part, and it was attenuated in deep regions due to the phenomenon known as aspect ratio-dependent scalloping attenuation (ARDSA) [22]. This phenomenon appears in narrow openings with high aspect ratios, and therefore it was observed only in the interior walls. The depth illuminated by the light was estimated to be between 60 µm and 70 µm, then the roughness was attenuated, and the scattering light loss was negligible within the intermediate walls. The only walls suffering from this scalloping were the external walls (first and last faces). From the SEM image in Figure 2a, the peak-to-bottom value of the scalloping was about 127.4 nm; this renders the root mean square roughness (σ) for these surfaces equal to about 45 nm. This value can be considered much less than the used light wavelength; besides, only two surfaces had this value, while σ was equal to almost zero for all the other surfaces. Thus, we believe the scattering losses can be considered negligible in our case.

The etching depth of the grooves into silicon was above 130 µm in order to be enough for hosting the FRLs and the measuring fibers of diameter about 125 µm with acceptable clearance for easy implementations into the chip. More details about this etching process can be found in Reference [22]. Fluidic inlet and outlet holes were opened by through-wafer etching. Then, the silicon chip was capped by glass substrate with an anodic bonding process. Figure 2b shows the fabrication flow starting from patterning a single crystal silicon wafer with thermal oxide, then transferring the structure into silicon by deep reactive ion etching (DRIE), and finally bonding of the glass capping substrate, after removing the oxide. After finishing the standard micromachining process and dicing the chips, next was the assembly step of inserting the input and output optical fibers from the two sides.

**Figure 3:**
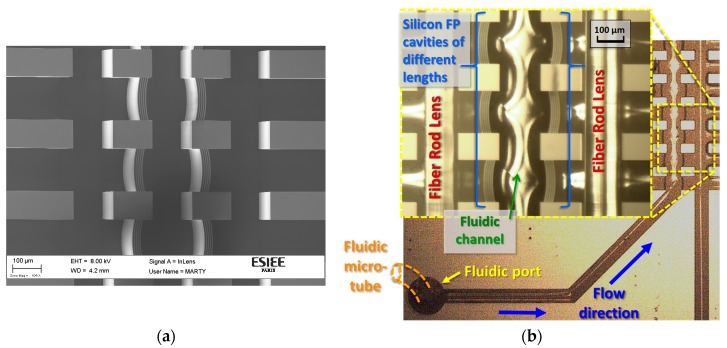
(**a**) An SEM image of the Bragg mirror depicting its high verticality and low roughness due to the well-controlled etching process. (**b**) Photo of the fabricated chip indicating how the external fluidic tubing is connected to the chip, and the fluid flow direction from the input port to the fluidic channel between the optical cavities. The inset is a zoom on the multi FP cavities.

After fabrication and dicing of the chips, stripped fibers forming the FRLs were placed into their grooves and the fluidic micro-tubes were fixed at the fluidic ports for delivering the liquid under test. Figure 3 shows the fabricated chip with multi FP cavities having different cavity lengths. Figure 3a shows an SEM image of the etched cavities and grooves on the chip before capping the glass substrate, and Figure 3b shows the final chip after inserting the FRLs.

For the optical measurements, a tunable laser source of model 81949A from Agilent (Agilent Technologies, Santa Clara, CA, USA) was used as the light source, and a power meter of model 81634B from Agilent was used as the detector; both were housed in the Agilent 8164B mainframe. That platform was equipped with a General Purpose Interface Bus (GPIB) interface to enable control by a PC for synchronizing the change of the injected light wavelength with the corresponding measured output power. Telecommunication single mode fibers were used for the connections, and cleaved fibers were used for injecting and collecting the light into and from the chip. The cleaved fibers were aligned into the chip grooves by two fiber positioners of five degrees of freedom, while the sample positioner was of two degrees of freedom.

## 4. Results

**Figure 4:**
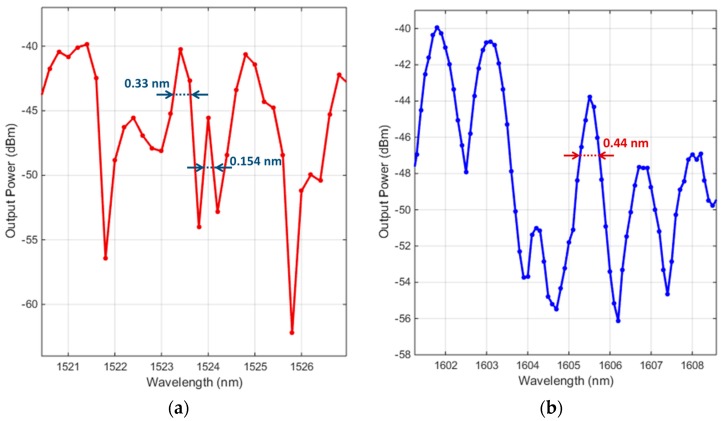
The spectrum for (**a**) ethanol (**b**) deionized (DI)-water filling a cavity of 318 µm physical length. Their sharp line widths are indicated, implying their high quality factor.

The measured spectra for a cavity with a physical length of 318 µm, filled ethanol and water, are presented in Figure 4a,b respectively.

The spectra in Figure 4 show several large peaks beside or interfering with smaller peaks. These large and small peaks correspond to fundamental and higher-order Hermite–Gaussian modes, respectively. The family of Hermite–Gaussian beams is the solution to Maxwell’s equations that take the form of narrow beams inside spherical-mirror resonators [21,23]. This also holds true with 1D and 2D curved-mirror resonators, as has been demonstrated experimentally and by numerical simulations [24]. The spectrum of ethanol shows noticeably more interference between the main peaks and the side peaks than the spectrum of DI-water. This is expected, as the interspacing between the fundamental and higher-order modes decreases with increasing refractive index of the analyte [14]. The refractive indices of ethanol and DI-water at the indicated wavelengths are 1.3507and 1.3169, respectively. In the case of ethanol, a superior *Q* factor of 9896 is proposed by a side mode of linewidth 0.154 nm. The main peak that has a linewidth of 0.33 nm, would provide a *Q* factor of 4616. In the case of DI-water, the modal interspacing is wider, and hence the modal interference is not significant, depicting better separated peaks. As depicted in Figure 4b, the quality factor in the case of DI-water could reach about 3649. It is normal to have lower *Q* factor in the case of a lossy liquid like water, as it has a non-negligible light absorption within that wavelength range.

**Figure 5:**
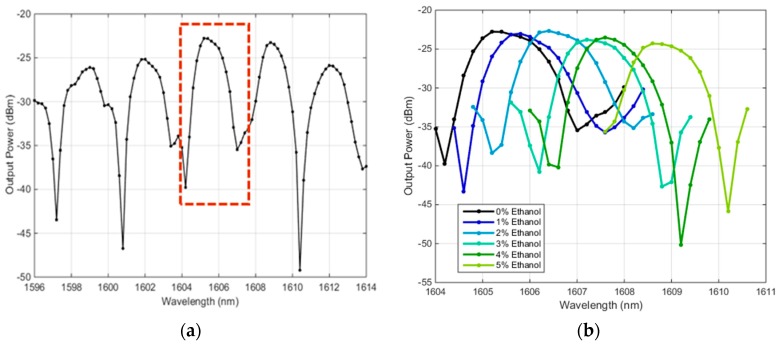
(**a**) The spectrum for DI-water for a cavity with 256 µm physical length. (**b**) The spectra for DI-water and mixtures of DI-water and ethanol with different ratios at the peak indicated in (**a**) by the dotted rectangle.

The sharp resonance peaks of the proposed resonator structure can provide good refractometry analyses for the analyte passing inside the resonator by tracing the peak wavelength shift happening when changing the liquid from a reference one. For a consistent refractometer behavior, selecting a spectral peak free from modal interference with side modes is preferred. Otherwise, the resonance peak wavelength will be altered due to modal coupling with changing analyte refractive index [14]. The selected resonance peak is indicated by the dotted rectangle in Figure 5a. The resonance wavelength of this peak shifts with changing mixing ratio of the ethanol–water mixture being measured, as shown in Figure 5b.

**Figure 6:**
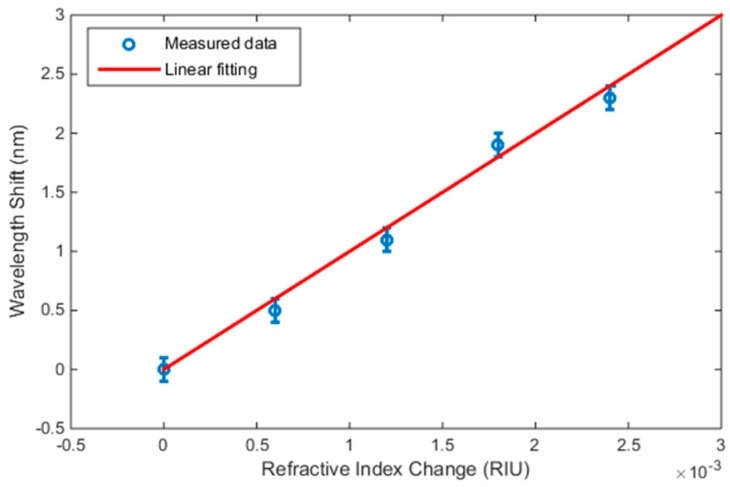
The resonance peak wavelength shift versus the refractive index of the analyte.

The values of the resonance peaks were obtained from Figure 5b. The refractive index values for the different mixing ratios were determined from literature [25]. Plotting the resonance peak shift *δλ* versus the corresponding change in refractive index *δn* is shown in Figure 6. The wavelength step used in the measurements was 0.2 nm; hence, the actual maxima may fall within ±0.1 nm from the allocated values. The error bars in Figure 6 were set to this value. The measurements were fitted by a linear line, whose slope determines the sensitivity *δλ*/*δn*, which is obtained to be 1000 nm/RIU. Up to our knowledge, this is the best sensitivity value obtained for a refractometer based on an on-chip FP cavity.

## 5. Conclusions

Curved surfaces can provide good electric field confinement for FP cavities. For easy fabrication on silicon chips, the curvatures for the parallel and perpendicular plans are decoupled. Cylindrical Bragg mirrors are used for providing the confinement in the plan parallel to the chip surface, while external FRLs reshape the light beam in the perpendicular plan before it enters the cavity. From the transmission spectra recorded from the cavity filled with liquids, a superior *Q* factor of 9896 for the side peak and 4616 for the main peak were attained in case of ethanol, which is an absorption-free liquid. An absorbing liquid can still have quite high *Q* factors; for example, DI-water attained a *Q* factor of about 3649. These are the highest *Q* factors reported for an on-chip FP cavity up to our knowledge. The proposed structure is used as an optofluidic refractometer on-chip. It can provide sensitivity up to 1000 nm/RIU, which to our knowledge is the highest attained sensitivity for a refractometer based on an on-chip FP cavity.
